# Supplementary material for: Impact of molecular symmetry on crystallization pathways in highly supersaturated KH2PO4 solutions
Source: Nat Commun. 2024 Apr 10;15:3117. doi: 10.1038/s41467-024-47503-1 (PMC11006877; doi:10.1038/s41467-024-47503-1)
Supplement: Supplementary file 1 — Supplementary Information [file 41467_2024_47503_MOESM1_ESM.pdf]

Supplementary Information: Impact of molecular symmetry on crystallization pathways in highly supersaturated KH_2PO_4 solutions

Yong Chan Cho[1], Sooheyong Lee[1,2], Lei Wang[1], Yun-Hee Lee[1], Seongheun Kim[3], Hyun-Hwi Lee[3], John Jonghyun Lee[4], Geun Woo Lee[1,2*]

[1] *Frontier of Extreme Physics, Korea Research Institute of Standards and Science, Daejeon 34113, Republic of Korea*

[2] *Applied Measurement Science, University of Science and Technology, Daejeon 34113, Republic of Korea*

[3] *Pohang Accelerator Laboratory, POSTECH, Pohang 37673, Republic of Korea*

[4] *Department of Mechanical Engineering, Iowa State University, Ames, IA 50011, United States of America*

***e-mail: gwlee@kriss.re.kr**

contents

Supplementary Note 1: The equations of the structure factor s(q) and PDF G(r) 2
Supplementary Note 2: The cluster-based reverse Monte-Carlo calculation for H2PO4[-] dimer 3
Supplementary Figure 1: The polar coordinates for PO_4 monomer and PO_4-PO_4 dimer 3
Supplementary Figure 2: Time dependence of the volume and supersaturation of the droplet. 5
Supplementary Figure 3: The measurement and calculation of droplet volume. 6
Supplementary Figure 4: The schematic diagram of in-situ X-ray and Raman scattering experiments on ESL system for aqueous solution droplet. 7
Supplementary Figure 5 μ-Raman spectra of KDP and ADP solution depending on the supersaturation. 8
Supplementary Table 1: Evolution of concentration, solution density, and n and α for the KDP solution droplet. 9
Supplementary Table 2: Evolution of concentration, solution density, and n and α for the ADP solution droplet. 10
Supplementary Table 3: Summary of the calculated results for the optimized $(H_2PO_4^-)_2$ dimeric unit block in KDP solution 11
Supplementary Table 4: Summary of the calculated results for the optimized $(H_2PO_4^-)_2$ dimeric unit block in ADP solution 12
Supplementary References 13

Supplementary Note 1: The equations of the structure factor s(q) and PDF G(r)

A general form of the X-ray intensity scattered coherently from samples in electron units ($I_{\mathrm{eu}}^{\mathrm{coh}}(q)$) is as below

$$I_{\mathrm{eu}}^{\mathrm{coh}}(q) = < f^2 > + < f >^2 \int_0^\infty 4\pi r^2[\rho(r) - \rho_0] \frac{\sin(qr)}{qr} \mathrm{d}r \quad (1)$$

Here, f is atomic form factor, $\rho(r)$ is the atomic number density, which is the mean weighted density of neighbor atoms at distance r from an atom at the origin, ρ_0 is the average atomic number density of the sample (a constant value at a given supersaturation), $q(= 4\pi \sin\theta/\lambda,\ 2\theta$ is the scattering angle) is the magnitude of the scattering momentum. Then, $s(q)$ is defined as

$$s(q) = \frac{I_{\mathrm{eu}}^{\mathrm{coh}}(q) - (<f^2> - <f>^2)}{<f>^2} \quad (2)$$

In general, pair distribution function (PDF) is denoted by $g(r)$ (=$\rho(r)/\rho_o$). In this study, what we used is the reduced PDF ($G(r) = 4\pi r(\rho(r) - \rho_o) = 4\pi r\rho_o (\rho(r)/\rho_o - 1) = 4\pi r \rho_o (g(r) - 1)$) [1].

The reduced pair distribution function (denoted by $G(r)$), is the truncated Fourier transform of the total scattering structure function ($s(q)$) as bellow

$$G(r) = \frac{2}{\pi} \int_{q_{\min}}^{q_{\max}} q(s(q) - 1) \sin(qr) \mathrm{d}q \quad (3)$$

, where $q(= 4\pi \sin\theta/\lambda,\ 2\theta$ is the scattering angle, λ is the scattering wavelength) is the magnitude of the scattering momentum, r is the distance from the center atom, $s(q)$ is the structure factor function. $G(r)$ gives higher oscillation intensity than g(r) in long distance, which distinctly shows changes in the oscillation. The $G(r)$ peaks are coming from actual interatomic distances in the material. And the radial distribution function is given as $4\pi r^2\rho(r)$ (=$4\pi r^2\rho_0 g(r)$). Therefore, the coordination number of the first neighbor atoms (between r_1 and r_2) was obtained in this research using

$$n = \int_{r_1}^{r_2} 4\pi r^2 \rho(r)\, \mathrm{d}r = \int_{r_1}^{r_2} (rG(r) + 4\pi r^2 \rho_0)\, \mathrm{d}r \quad (4)$$

Supplementary Note 2: The cluster-based Reverse Monte-Carlo calculation for $H_2PO_4^-$ dimer

1. Calculation of Δs(q) from experimentally obtained s(q): Coherent scattering from the supersaturated KDP and ADP solution is mostly contributed by the $H_2PO_4^-$ molecules since they are likely the most well-ordered structures in these solutions. We expect relatively strong coherent scattering from the $H_2PO_4^-$ molecules, especially in a higher supersaturated solution. It is also assumed that the cations (K^+ or NH_4^+) are randomly distributed in the solution[2-5]. Therefore, the change of the Δ*I*(*q*) for solutes can be obtained from experimentally obtained I(q) by Eq. 5

$$\Delta I(q) = I(q)_{\text{solution}} - \alpha \times I(q)_{\text{water}} \tag{5}$$

Here, the α indicates scattering ratio from water molecules in the solution (see also Supplementary Tables 1 and 2). Using the experimentally obtained Δ*I*(*q*), the Δ*s*(*q*) and the Δ*G*(*r*) were obtained by Pdfgetx2 program.

2. Coordination of $(H_2PO_4^-)_2$ dimeric unit block model: Atomic positions for P and O_p are given in spherical coordinates (Supplementary Fig. 1). For the monomer, the atomic coordinates of a PO_4 tetrahedron are set by spherical coordinates (Supplementary Fig. 1a). The dimer is composed by two monomers. One monomer is placed at origin and the other monomer is placed with a distance from origin (Supplementary Fig. 1b).

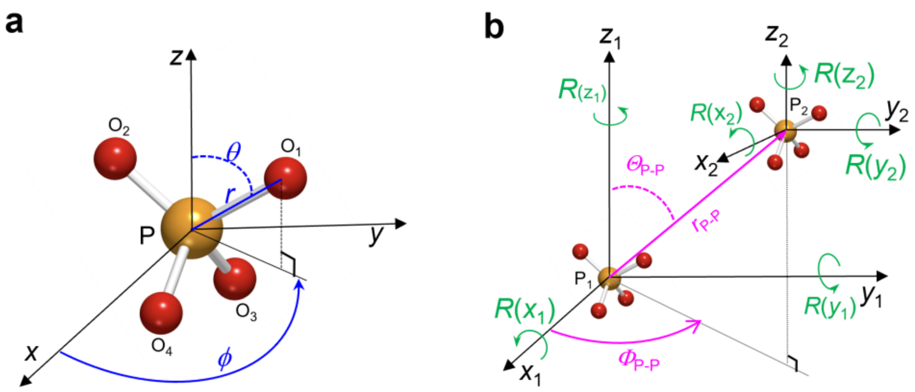

Supplementary Figure 1: The polar coordinates for PO_4 monomer and PO_4-PO_4 dimer in this study. a spherical coordinate for a H_2PO_4 monomer (H atom is omitted), A phosphorus atom (orange sphere) is placed at origin and the coordination of 4 oxygen atoms (red sphere) are presented by the spherical coordinates (r, θ, φ) (blue color). **b** spherical coordinates for dimer composed of two monomers. The P_1 is placed at origin (0,0,0) and the position of P_2 is described by using spherical coordinates ($r_{P\text{-}P}$, $\Theta_{P\text{-}P}$, $\Phi_{P\text{-}P}$) (magenta color). The rotation ($R(x)$, $R(y)$, $R(z)$) (orange) operation is applied to both two $H_2PO_4^-$ monomers.

3. Calculation of s_{cal} for cluster modeling of $H_2PO_4^-$: The $s_{cal}(q)$ for the $H_2PO_4^-$ molecule model is calculated using Eq. 6 [6-8],

$$s_{\mathrm{cal}}(q) = \left(\frac{1}{\sum_{j=1}^{N} f_j^2} \sum_{j=1}^{N} \sum_{k=1}^{N} f_j f_k \frac{\sin(q\, r_{jk})}{q\, r_{jk}} - 1\right) \times \mathrm{e}^{-2\mathrm{DWF}\times q^2} + 1 \qquad (6)$$

where N is the number of particles, q is wavevector, DWF is Debye Waller factor, f_j, f_k are atomic form factors, r_{jk} is atomic distance between j^{th} and k^{th} atoms. Previously, this method was successfully applied to explain SRO in liquid metals[6-8]. In this study, $\underline{s_{cal}}(q)$ is calculated to fit the experimentally obtained $\Delta s(q)$ within the q ranges (3.5 Å^{-1}~ 8.5 Å^{-1}) with changing the symmetry and geometry of monomer and dimer. In this study, the deviation between s_{cal} and $\Delta s(q)$ is minimized by using non-linear least-squares minimization and curve-fitting routine provided in Python program language (LMFIT package). Within the constraint condition for its symmetry, the geometry parameters of two monomers and Debye-Weller factors were optimized up to minimize χ^2, which is given in Eq. 7.

$$\chi^2 = \sum_i^N (s_{\mathrm{cal}}(q_i) - \Delta s(q_i))^2 \qquad (7)$$

Supplementary Table 3 and Supplementary Table 4 present the summary of calculated results for KDP and ADP solution, respectively. In the dilute KDP and ADP solutions, the $H_2PO_4^-$ anion has the C_{2v} molecular symmetry which is used to set the initial constraint conditions with r, θ and φ (see Supplementary Figure 1). In this calculation, we fix φ values of those of a right tetrahedron. The constraint for the P-O distance is given by the FWHM of the G1 peak of Fig. 3 in the main text. The calculated $s_{cal}(q)$ is fitted to the experimentally obtained structure factor of solute ($\Delta s(q)$) between q=3.5Å^{-1} and 8.5 Å^{-1}. The constrain condition for its symmetry is provided by changing P-O distances or/with O-P-O angles. The initial symmetry for PO_4 is C_{2v} (type 1 presented in Fig. 4b in the main text). The initial dimer is composed of two same geometry monomers (SM) with C_{2v} symmetry (SM-C_{2v}). The C_s symmetry has only one mirror symmetry (σ_v) by breaking 2- fold symmetry. Subsequently, the breakage of the σ_v symmetry results in C_1. Depending on the symmetry of monomer (C_{2v}, C_s, C_1) and geometry condition (same monomer (SM) of different monomer (DM)), the suitable constraint conditions are used. The models are sequentially tested for SM-C_s (the dimer with SM with C_s symmetry), DM-C_s (the dimer composed of DM with C_s symmetry) and finally DM-C_1 (the dimer with DM with C_1 symmetry). In this calculation, we accepted the model having the least deviation ($\chi^2 <$ 0.05). After calculation, the result of unphysical dimer geometry (e.g., too close O-O and P-O distance) was excluded. Here, hydrogen atoms are omitted in this calculation.

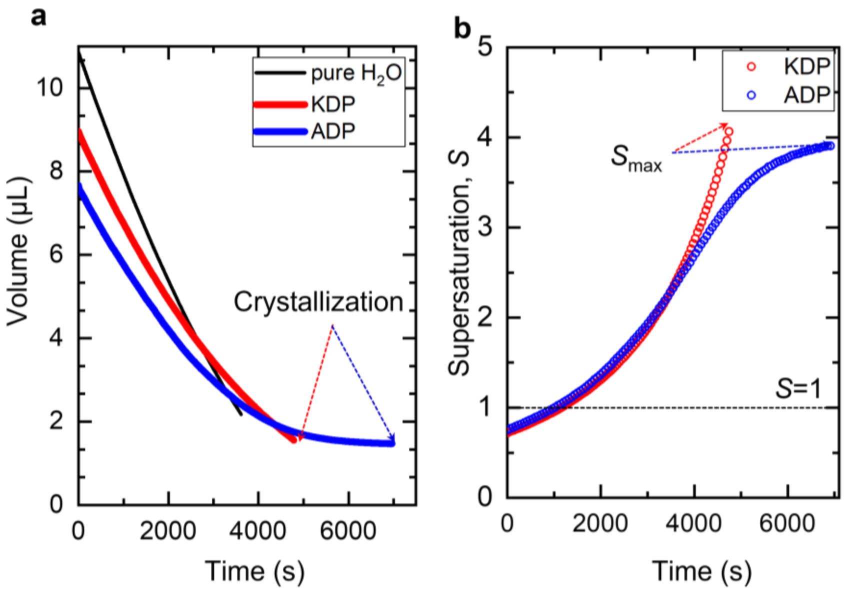

Supplementary Figure 2: Time dependence of the volume and supersaturation of the droplet. The concentration of the levitated solution droplet increases by evaporation and approaches the highest supersaturation values for more than 4500 s. In this experiment, we take images of the levitated solution droplet every 5 seconds. When the solution crystallizes at a supersaturation, we consider the supersaturation just before the crystallization event. Since the evaporation rate is much slow, the change of supersaturation for 5 seconds is negligible. **a** The decrease of droplet volume depending on the levitation time. The dashed arrows indicate the occurrence of crystallization events. The droplet volume was obtained by the Legendre polynomial fitting from the detected droplet edge. **b** The increase of supersaturation of droplet with increasing time. The dashed arrows indicate the maximal supersaturation points (S_{max}) just before the crystallization event. S=1 is presented for guiding the solubility limits of KDP and ADP solutions. Source data are provided as a Source Data file.

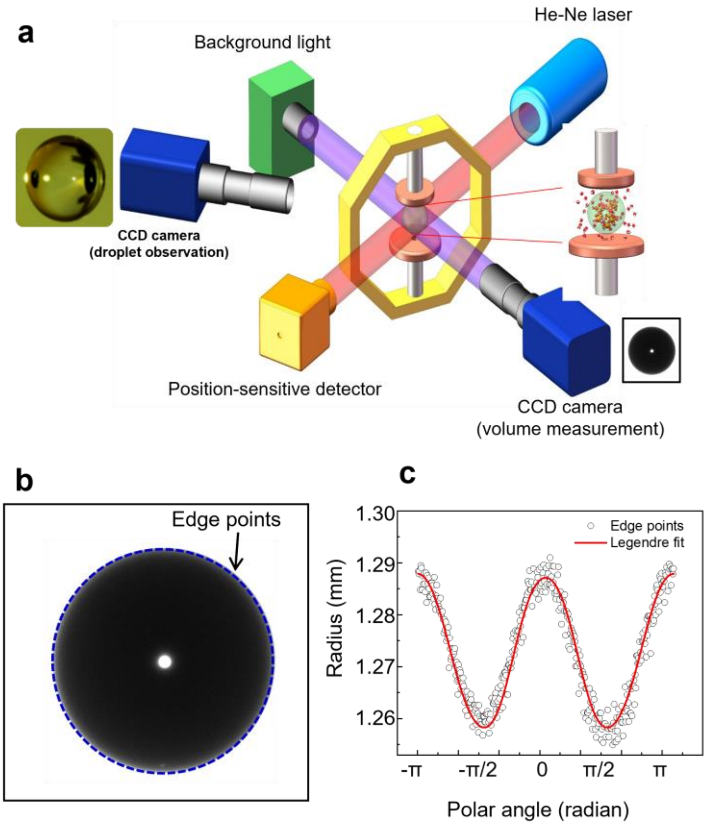

Supplementary Figure 3: The measurement and calculation of droplet volume. In order to determine the radius of the solution droplet, we used a differential contrast method[9] which determines the edge detection of the shadow images. The detected edge points in Cartesian coordinates are transformed into polar coordinates and then fitted with the 6th-order Legendre polynomial, $(R(\theta) = \sum_{l=0}^{6} c_n P_l(\cos(\theta)))$, where P_l (cos(θ)) is the l-th order Legendre polynomial and c_n is the coefficients determined by the 6th order Legendre polynomial fitting. The droplet volume (V) is obtained by integrating the fitting results according to $V = (2\pi/3)\int_{\pi}^{0} R(\theta)^3 \sin(\theta) \mathrm{d}\theta$. Finally, we can determine the concentration from the solute mass and solution volume, then the supersaturation is given from the definition, $S = C_s/C_e$. **a** The schematic diagram for the volume measurement using B/W CCD camera and the droplet observation using the other CCD camera. **b** The snapshot of the droplet shadow image and the edge-detection. **c** The volume calculation by applying the 6th-order Legendre polynomials fitting.

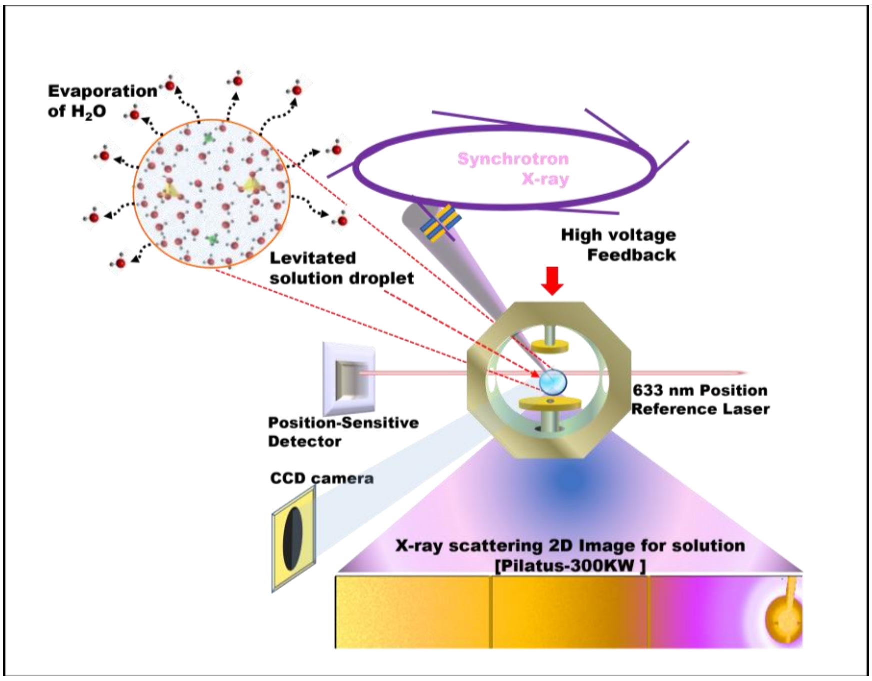

Supplementary Figure 4: The schematic diagram of in-situ X-ray and Raman scattering experiments on ESL system for aqueous solution droplet. The ESL components are installed at Pohang Light Source II (PLS II) (Suppl. Fig. 3), which is used to measure the X-ray scattering pattern in stable and metastable states. *In-situ* X-ray scattering experiment is performed at the undulator insertion device beamline at Pohang Light Source II (BL5A and 1C)[10-12]. Monochromatized 18 keV X-ray from a cryogenically cooled silicon (111) double crystal monochromator is delivered to the sample chamber through a delivery-pipe filed with helium gas. The X-ray detector (Pilatus-300KW, 1475 x 195 pixels with a pixel size of 172 μm x 172 μm) is just placed behind the ESL chamber to collect the scattered signals from the solution droplet. Only when the supersaturation of droplet reaches at the targeted value, X-ray scattering X-ray beam is irradiated on droplet and the scattering 2D image is recorded on Pilatus-300KW. The conversion of the 2D image to 1D data was carried out by using Fit2D and Dioptas programs. Subsequently, the $s(q)$ and $G(r)$ were obtained by using Pdfgetx2 program.

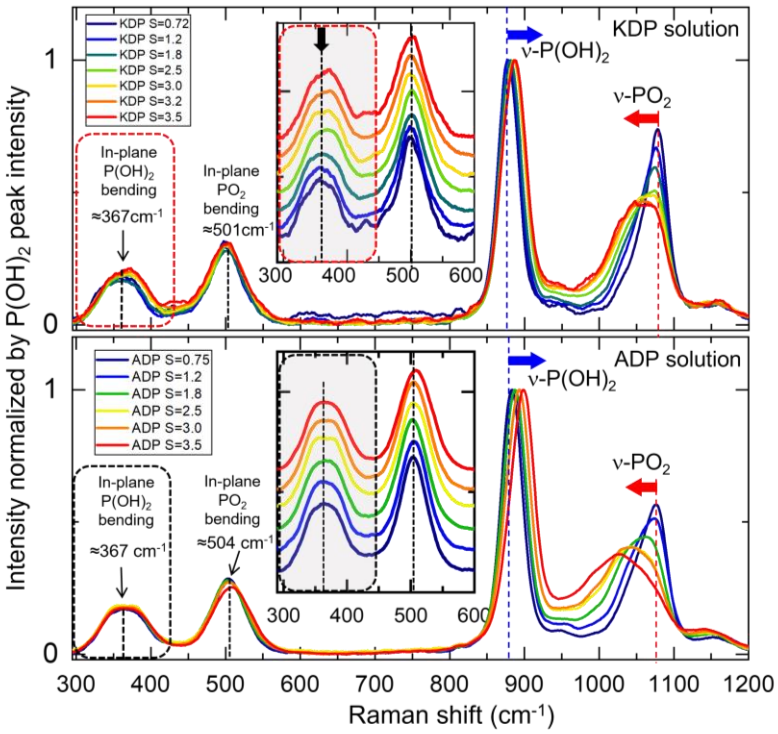

Supplementary Figure 5: micro-Raman spectra of KDP and ADP solution depending on the supersaturation. An incident laser (wavelength of 532 nm) focused on the levitated solution drop. After reaching the targeted supersaturation level, the measurement starts with the irradiation of laser on levitated droplet. Every spectrum of different supersaturation level was measured independently instead of keeping continuous measurement for one droplet. The large peaks near 870 cm^{-1} and 1070 cm^{-1} are corresponding to the stretching mode of $P(OH)_2$ and PO_2. The peak shift of the stretching mode (the blue arrow (the blue-shift of v-$P(OH)_2$) and red arrows (the red-shift of v-PO_2) with increasing supersaturation agree with those of the previous report[13]. The peaks between 300 cm^{-1} - 600 cm^{-1} are corresponding to in-plane O-P-O bending deformation mode[13]. In particular, we find a change of the peak in 300 cm^{-1} - 450 cm^{-1} (the red box with dashed line in Figure) which corresponds to the two in-plane $P(OH)_2$ bending deformation mode. For KDP solutions, the peak for in-plane $P(OH)_2$ changes to an asymmetric shape (marked by a black arrow near 367 cm^{-1}) with increasing supersaturation, while no change is observed in ADP solution. Although the peak is observed in another study at low supersaturated solution[14] which is the same as our measurement, such an asymmetric shape was not reported. This reflects the change of intra-structure of the $H_2PO_4^-$ in KDP solution, but not in ADP solution. Source data are provided as a Source Data file.

Supplementary Table 1: **Evolution of concentration (*c* (KDP mass (g) / H_2O volume(cm^3)) and *M*(molarity)), solution density, the number of water molecules per solute ion (*n*), the number ratio of water molecules to total solution atoms (α) for the KDP solution droplet.** The solubility limit of the KDP solution (*S*=1) is 25 g per 100mL. The α is obtained by calculating the number ratio of water molecules to total atoms. For KDP, each solute ion has 21 water molecules (at *S*=*1*) on average. Therefore, the α (at *S*=1) is given by 0.87 (≈ 42 / (6+42), where 6 comes from the number of atoms in solute molecules (i.e., K and PO_4). Here, hydrogen atoms are omitted because of their negligible scattering by X-ray.

Volume contraction Ratio	Concentration			solution density (g cm^{-3})	The number of H_2O per ion (*n*)	The number ratio of water to solution atoms for calculation of X-ray scattering (except hydrogen atoms) (α)
V(*t*)/*V*(*t*=0)	Initial concentration *m*(KDP)/*V*(Water) (g cm^{-3})	molarity *M* (mol L^{-1})	Supersaturation (*S*)			
1	0.18	1.240	0.72	1.11	21.0	0.87
0.60		2.066	1.2	1.18	12.1	0.80
0.48		2.583	1.5	1.22	9.4	0.76
0.36		3.444	2	1.30	6.7	0.69
0.29		4.305	2.5	1.38	5.1	0.63
0.23		5.510	3.2	1.48	3.7	0.55
0.22		5.682	3.3	1.50	3.5	0.54
0.21		6.027	3.5	1.53	3.3	0.52
0.18		7.060	4.1	1.62	2.6	0.46

Supplementary Table 2: Evolution of concentration (*c* (ADP mass (g) / H_2O volume(cm^3)) and *M*(molarity)), solution density, the number of water molecules per solute ion (*n*), the number ratio of water molecules to total solution atoms (α) for the ADP solution droplet. The solubility limit of the ADP solution (S=1) is 40 g per 100mL. For ADP, each solute ion has 10.7 water molecules (at S=1) in average. Therefore, the α (at S=1) is given by 0.78 (≈ 21.4 / (6+21.4), where 6 comes from the number of atoms in solute molecules (i.e., N and PO_4). Here, hydrogen atoms are omitted because of their negligible scattering by X-ray.

Volume contraction Ratio	Concentration			solution density (g cm^{-3})	The number of H_2O per ion (n)	The number ratio of water to solution atoms for calculation of X-ray scattering (except hydrogen atoms) (α)
$V(t)/V(t=0)$	Initial concentration m(KDP)/V(Water) (g cm^{-3})	molarity M (mol L^{-1})	Supersaturation (S)			
**1**		2.271	0.75	1.13	10.7	0.78
**0.63**		3.634	1.2	1.21	6.1	0.67
**0.42**		5.451	1.8	1.32	3.6	0.54
**0.34**		6.662	2.2	1.39	2.6	0.47
**0.31**		7.268	2.4	1.43	2.3	0.43
**0.29**	0.30	7.873	2.6	1.47	2.0	0.40
**0.27**		8.479	2.8	1.50	1.7	0.36
**0.25**		9.084	3.0	1.54	1.5	0.33
**0.23**		9.690	3.2	1.57	1.3	0.30
**0.22**		10.296	3.4	1.61	1.2	0.28
**0.21**		10.901	3.6	1.65	1.0	0.25

Supplementary Table 3: Summary of the calculated results for the optimized $(H_2PO_4^-)_2$ dimeric unit block in KDP solution: The light-yellow color presents the dimer composed of two same monomers with C_{2v} symmetry (SM-C_{2v}). The dark-yellow color presents the dimer composed of two same monomers with C_s symmetry (SM-C_s). The blue color depicts the dimer composed of different monomers with C_1 symmetry (SM-C_1). Comparison between the optimized results is presented by the dark gray color.

KDP *S* (*n*)	Symmetry of monomers	Dimer geometry				DWF	χ^2
		P-O_P (Å)	θ (degree)	P-P distance (Å)	Ave. O_P-O_P (Å)		
0.72(21.0)	SM-C_{2v}	r_1=r_2=1.30, r_3=r_4=1.70	θ_1=θ_2=51.75, θ_3=θ_4=125.6	3.80	2.46	0.0169	0.0463
1.2 (12.1)	SM- C_{2v}	r_1=r_2=1.30, r_3=r_4=1.70	θ_1= θ_2=52.14, θ_3= θ_4=125.28	3.84	2.45	0.0172	0.0208
1.5 (9.4)	SM-C_s	r_1=r_2 =1.30, r_3=r_4=1.70	θ_1=θ_2=60.26, θ_3=132.4, θ_4=121.23	3.63	2.45	0.0169	0.0374
2.0 (6.7)	SM-C_s	r_1=r_2=1.30, r_3=r_4=1.70	θ_1= θ_2=57.9, θ_3=123.74, θ_4=133.25	3.63	2.45	0.0182	0.0069
2.5 (5.1)	SM-C_s	r_1=r_2=1.30, r_3=r_4=1.70	θ_1= θ_2=53.94, θ_3=124.15, θ_4=133.25	3.64	2.45	0.0179	0.0058
3.2 (3.7)	SM-C_s	r_1=r_2=1.50, r_3=1.30, r_4=1.70	θ_1= θ_2=51.75, θ_3=127.88, θ_4=129.68	3.71	2.45	0.0201	0.0800
	DM-Cs	r_1=r_2=1.70, r_3=r_4=1.30	θ_1= θ_2=51.75 θ_3=133.25, θ_4=124.42	3.70	2.47	0.0108	0.0369
		r_1= r_2 =1.53, r_3=1.30, r_4=1.70	θ_1= θ_2=51.75, θ_3=128.25, θ_4= 130.47				
3.3 (3.5)	SM-C_s	r_1= r_2 =1.45, r_3 =1.30, r_4=1.70	θ_1= θ_2=51.75, θ_3=123.99, θ_4=132.85	3.71	2.41	0.0203	0.0953
	DM-C_s	r_1= r_2=1.30, r_3=1.70, r_4=1.30	θ_1= θ_2=51.75, θ_3=133.25, θ_4=122.56	3.71	2.44	0.0160	0.0368
		r_1= r_2=1.70, r_3=1.30, r_4=1.62	θ_1=θ_2=51.75, θ_3 =131.65, θ_4 =126.61				
	SM-C_1	r_1= r_2=1.70, r_3=1.31, r_4 =1.70	θ_1= θ_2=51.75, θ_3=133.24, θ_4=124.01	3.70	2.46	0.0154	0.0642
	DM-C_1	r_1= r_2=1.30, r_3=1.70, r_4=1.30	θ_1=51.75, θ_2=53.85, θ_3=133.25, θ_4=123.62	3.71	2.44	0.0156	0.0367
		r_1=1.70, r_2=1.7, r_3=1.63, r_4=1.30	θ_1=51.75, θ_2=51.85, θ_3=130.4, θ_4=127.56				
3.5 (3.3)	SM-C_s	Could not fitted. [the result gives only Non-physical situation]					
	DM-C_s	r_1 = r_2 = 1.36, r_3 = r_4 = 1.30	θ_1=θ_2=53.75, θ_3=133.25, θ_4=127.29	3.70	2.40	0.017	0.0525
		r_1 = r_2 = 1.69, r_3= 1.67 r_4=1.38	θ_1=θ_2=51.75, θ_3=130.08, θ_4=129.52				
	SM-C_1	r_1=1.3, r_2=1.7, r_3=1.31, r_4 = 1.7	θ_1=51.76, θ_2=53.29, θ_3=133.25, θ_4=127.37	3.70	2.46	0.0154	0.0518
	DM-C_1	r_1 = 1.30, r_2 = 1.31 r_3= 1.70, r_4 = 1.71	θ_1=51.75, θ_2=56.49 θ_3=133.25, θ_4=127.36	3.70	2.45	0.0155	0.0213
		r_1 = 1.30, r_2 = 1.70 r_3 = 1.30, r_4 = 1.70	θ_1=51.75, θ_2=51.75 θ_3=129.11, θ_4=130.53				
4.1 (2.6)	DM-C_1	r_1 = 1.30, r_2 = 1.32 r_3= 1.68, r_4=1.70	θ_1=51.75, θ_2=51.75 θ_3=133.25, θ_4=123.87	4.2	2.47	0.0163	0.0306
		r_1 = 1.30, r_2 = 1.43 r_3 = 1.69, r_4 = 1.68	θ_1=51.75, θ_2=51.75 θ_3=133.25, θ_4=132.99				

Supplementary Table 4: Summary of the calculated results for the optimized $(H_2PO_4^-)_2$ dimeric unit block in ADP solution: The light-yellow color presents the dimer composed of two same monomers with C_{2v} symmetry (SM-C_{2v}).

ADP S (n)	Symmetry of Monomers	Dimer geometry				DWF	χ^2
		P-O_P (Å)	θ (degree)	P-P (Å)	Ave. O_P-O_P (Å)		
0.75(10.7)	SM-C_{2v}	r_1=r_2=1.30, r_3=r_4=1.70	θ_1 = θ_2 =51.75, θ_3=θ_4=130.37	3.68	2.45	0.0077	0.0391
1.2(6.1)	SM-C_{2v}	r_1=r_2 =1.30, r_3=r_4=1.69	θ_1=θ_2=51.75, θ_3=θ_4=129.12	3.62	2.45	0.0121	0.0175
1.8(3.6)	SM-C_{2v}	r_1=r_2=1.32, r_3=r_4=1.67	θ_1=θ_2=51.75, θ_3=θ_4=129.72	3.83	2.46	0.0154	0.0412
2.2(2.6)	SM-C_{2v}	r_1=r_2=1.32, r_3=r_4=1.69	θ_1=θ_2=51.75, θ_3=θ_4=131.35	3.82	2.45	0.0128	0.0338
2.4(2.3)	SM-C_{2v}	r_1=r_2=1.32, r_3=r_4=1.68	θ_1=θ_2=51.75, θ_3=θ_4=133.25	4.00	2.45	0.0128	0.0369
2.6(2.0)	SM-C_{2v}	r_1=r_2=1.30, r_3=r_4=1.69	θ_1=θ_2=51.75, θ_3=θ_4=133.25	4.02	2.44	0.0122	0.0283
2.8(1.7)	SM-C_{2v}	r_1=r_2=1.30, r_3=r_4=1.69	θ_1=θ_2=51.75, θ_3=θ_4=133.25	4.00	2.44	0.0125	0.0293
3.0(1.5)	SM-C_{2v}	r_1=r_2=1.30, r_3=r_4=1.69	θ_1=θ_2=51.75, θ_3=θ_4=133.25	3.96	2.44	0.0125	0.0319
3.2(1.3)	SM-C_{2v}	r_1=r_2=1.30, r_3=r_4=1.69	θ_1=θ_2=51.75, θ_3=θ_4=133.25	3.95	2.44	0.0124	0.0253
3.4(1.2)	SM-C_{2v}	r_1=r_2=1.30, r_3=r_4=1.69	θ_1=θ_2=51.75, θ_3=θ_4=133.16	3.95	2.44	0.0128	0.0222
3.6(1.0)	SM-C_{2v}	r_1=r_2=1.30, r_3= r_4=1.69	θ_1=θ_2=51.75, θ_3=θ_4=133.05	3.95	2.44	0.0134	0.0204

Supplementary references

1 Waseda. Y, The Structure of Non-Crystalline Materials: Liquids and Amorphous Solids. McGraw-Hill, New York McGraw-Hill International Book Company (1980)

2. Preston, C. M. & Adams, W. A. A laser Raman spectroscopic study of aqueous orthophosphate salts. *J. Phys. Chem* **83**, 814–821 (1979).

3. Rudolph, W. W. Raman- and infrared-spectroscopic investigations of dilute aqueous phosphoric acid solutions. *Dalton Trans.* **39**, 9642–9653 (2010).

4. Syed, K. A., Pang, S.-F., Zhang, Y. & Zhang, Y.-H. Micro-Raman observation on the H2PO4− association structures in a supersaturated droplet of potassium dihydrogen phosphate (KH2PO4). *J Chem Phys* **138**, 024901 (2013).

5. Lu, G. W. et al. Cluster Formation in Solid–Liquid Interface Boundary Layers of KDP Studied by Raman Spectroscopy. *Phys Status Solidi* **188**, 1071–1076 (2001).

6. Lee, G. W., Gangopadhyay, A. K., Hyers, R. W., Rathz, T. J., Rogers, J. R., Robinson, D. S., Goldman, A. I. & Kelton, K. F. Local structure of equilibrium and supercooled Ti-Zr-Ni liquids, *Phys. Rev. B* **77,** 184102 (2008)

7. Lee, G. W., Gangopadhyay, A. K., Kelton, K. F., Hyers, R.W., Rathz, T. J., Rogers J. R., and Robinson, D. S. Difference in Icosahedral Short-Range Order in Early and Late Transition Metal Liquids, *Phys. Rev. Lett.* **93,** 037802 (2003)

8. Paradis, P. -F., Ishikawa, T., Lee, G. W., Holland-Moritz, D., Brillo, J., Rhim, W. -K., Okada, J. T. Materials properties measurements and particle beam interactions studies using electrostatic levitation. *Mater. Sci. Eng. R* **76,** 1–53 (2014).

9. Yoo, H. B., Park, C., Jeon, S. Lee, S., Lee, G. W. Uncertainty evaluation for density measurements of molten Ni, Zr, Nb and Hf by using a containerless method. *Metrologia* **52,** 677-684 (2016).

10. Lee, S. et al. Multiple pathways of crystal nucleation in an extremely supersaturated aqueous potassium dihydrogen phosphate (KDP) solution droplet. *Proc. Nat. Acad. Sci. USA* **113,** 13618-13623 (2016).

11 Hwang, H. *et al.* Hydration breaking and chemical ordering in a levitated NaCl solution droplet beyond metastable zone width limit: Evidence for early stage of the two-step nucleation. *Chem. Sci.* **12,** 179-187 (2021)

12 Lee, S., Jo, W., Cho, Y. C., Lee, H. H. & Lee, G. W. Solution electrostatic levitator for measuring surface properties and bulk structures of an extremely supersaturated solution drop above metastable zone width limit. *Rev. Sci. Instrum.* **88,** 055101 (2017).

13. Lu, G.W., and SUN, X., Raman Study of Lattice Vibration Modes and Growth Mechanism of KDP Single Crystals, *Cryst. Res. Technol.* 37 93-99 (2002)

14. Sun, C., Chen, X., Xue, D., Hydrogen Bonding Paradigm in the Formation of Crystalline KH2PO4 from Aqueous Solution, *Cryst. Growth Des*. **17**, 3178–3184 (2017)
