# Supplementary material for: Impact of molecular symmetry on crystallization pathways in highly supersaturated KH2PO4 solutions
Source: Nat Commun. 2024 Apr 10;15:3117. doi: 10.1038/s41467-024-47503-1 (PMC11006877; doi:10.1038/s41467-024-47503-1)
Supplement: Supplementary file 3 — Description of Additional Supplementary Files [file 41467_2024_47503_MOESM3_ESM.pdf]

Description of Additional Supplementary Files

File Name: Supplementary Movie 1

Description: Rapid crystallization of ADP solution. The video shows the rapid crystallization of the highly supersaturated ADP solution of S ≈ 3.5, which is corresponding to n (number of H2O per ion) ≈ 1.1.
