# Supplementary material for: Impact of molecular symmetry on crystallization pathways in highly supersaturated KH2PO4 solutions
Source: Nat Commun. 2024 Apr 10;15:3117. doi: 10.1038/s41467-024-47503-1 (PMC11006877; doi:10.1038/s41467-024-47503-1)
Supplement: Supplementary file 5 — Reporting Summary [file 41467_2024_47503_MOESM5_ESM.pdf]

nature portfolio

Corresponding author(s): Geun Woo Lee

Last updated by author(s): Feb 23, 2024

Reporting Summary

Nature Portfolio wishes to improve the reproducibility of the work that we publish. This form provides structure for consistency and transparency in reporting. For further information on Nature Portfolio policies, see our Editorial Policies and the Editorial Policy Checklist.

Statistics

For all statistical analyses, confirm that the following items are present in the figure legend, table legend, main text, or Methods section.

n/a	Confirmed	
☐	☒	The exact sample size (*n*) for each experimental group/condition, given as a discrete number and unit of measurement
☐	☒	A statement on whether measurements were taken from distinct samples or whether the same sample was measured repeatedly
☐	☒	The statistical test(s) used AND whether they are one- or two-sided<br>*Only common tests should be described solely by name; describe more complex techniques in the Methods section.*
☐	☒	A description of all covariates tested
☐	☒	A description of any assumptions or corrections, such as tests of normality and adjustment for multiple comparisons
☐	☒	A full description of the statistical parameters including central tendency (e.g. means) or other basic estimates (e.g. regression coefficient) AND variation (e.g. standard deviation) or associated estimates of uncertainty (e.g. confidence intervals)
☒	☐	For null hypothesis testing, the test statistic (e.g. *F*, *t*, *r*) with confidence intervals, effect sizes, degrees of freedom and *P* value noted<br>*Give P values as exact values whenever suitable.*
☒	☐	For Bayesian analysis, information on the choice of priors and Markov chain Monte Carlo settings
☒	☐	For hierarchical and complex designs, identification of the appropriate level for tests and full reporting of outcomes
☒	☐	Estimates of effect sizes (e.g. Cohen's *d*, Pearson's *r*), indicating how they were calculated

Our web collection on statistics for biologists contains articles on many of the points above.

Software and code

Policy information about availability of computer code

Data collection	x-ray scattering (pillatus-300kW Dectris operation program, TVX), Raman scattering (Andor Solis 4-30)
Data analysis	x-ray intensity calibration (Dioptas 5.0), conversion of s(q) and G(r) (PDF-getx2), Data plot (OriginPro 2018), calculation Sq dimer (python 3.0), Figure presentation (MS office power point)

For manuscripts utilizing custom algorithms or software that are central to the research but not yet described in published literature, software must be made available to editors and reviewers. We strongly encourage code deposition in a community repository (e.g. GitHub). See the Nature Portfolio guidelines for submitting code & software for further information.

Data

Policy information about availability of data

All manuscripts must include a data availability statement. This statement should provide the following information, where applicable:

- Accession codes, unique identifiers, or web links for publicly available datasets
- A description of any restrictions on data availability
- For clinical datasets or third party data, please ensure that the statement adheres to our policy

All relevant data are included in this article and its Supplementary Information files. The data that support the findings of this study are available from the corresponding authors Geun Woo Lee upon request. Source data are provided with this paper

Research involving human participants, their data, or biological material

Policy information about studies with human participants or human data. See also policy information about sex, gender (identity/presentation), and sexual orientation and race, ethnicity and racism.

Reporting on sex and gender	*Use the terms sex (biological attribute) and gender (shaped by social and cultural circumstances) carefully in order to avoid confusing both terms. Indicate if findings apply to only one sex or gender; describe whether sex and gender were considered in study design; whether sex and/or gender was determined based on self-reporting or assigned and methods used. Provide in the source data disaggregated sex and gender data, where this information has been collected, and if consent has been obtained for sharing of individual-level data; provide overall numbers in this Reporting Summary. Please state if this information has not been collected. Report sex- and gender-based analyses where performed, justify reasons for lack of sex- and gender-based analysis.*
Reporting on race, ethnicity, or other socially relevant groupings	*Please specify the socially constructed or socially relevant categorization variable(s) used in your manuscript and explain why they were used. Please note that such variables should not be used as proxies for other socially constructed/relevant variables (for example, race or ethnicity should not be used as a proxy for socioeconomic status). Provide clear definitions of the relevant terms used, how they were provided (by the participants/respondents, the researchers, or third parties), and the method(s) used to classify people into the different categories (e.g. self-report, census or administrative data, social media data, etc.) Please provide details about how you controlled for confounding variables in your analyses.*
Population characteristics	*Describe the covariate-relevant population characteristics of the human research participants (e.g. age, genotypic information, past and current diagnosis and treatment categories). If you filled out the behavioural & social sciences study design questions and have nothing to add here, write "See above."*
Recruitment	*Describe how participants were recruited. Outline any potential self-selection bias or other biases that may be present and how these are likely to impact results.*
Ethics oversight	*Identify the organization(s) that approved the study protocol.*

Note that full information on the approval of the study protocol must also be provided in the manuscript.

Field-specific reporting

Please select the one below that is the best fit for your research. If you are not sure, read the appropriate sections before making your selection.

☐ Life sciences ☐ Behavioural & social sciences ☒ Ecological, evolutionary & environmental sciences

For a reference copy of the document with all sections, see nature.com/documents/nr-reporting-summary-flat.pdf

Ecological, evolutionary & environmental sciences study design

All studies must disclose on these points even when the disclosure is negative.

Study description	We observed that the supersaturated KDP solution took the multiple pathways of crystallization through an intermediate metastable crystalline phase, depending on the degree of supersaturation. In contrast, ADP solution did not show such behavior although they have the same crystal structure. We try to understand the behind mechanism from microscopic viewpoint in this study by combining electrostatic levitation (ESL) with synchrotron X-ray scattering. Based on the synchrotron data and cluster modeling, we found the solution-solution transition, caused by changing the molecular symmetry of the solute from C2v to C1 occurred in the KDP solution as supersaturation increases. Therefore, we build a connection between the structural evolution (molecular symmetry) and the phase selection.
Research sample	KH2PO4 (KDP) and NH4H2PO4 (ADP) aqueous solutions, prepared by dissolving KDP crystal of purity 99.99% (Aldrich INC) and ADP crystal of purity 99.9% (Aldrich INC) in deionized water.
Sampling strategy	In this study, the determination of supersaturation (S) and the liquid structure at various supersaturations are the most important. The supersaturation is derived from droplet volume measured by well-developed imaging method. For the crystallization behavior at various supersaturation, statistical measurements are employed (i.e., the probability distribution of the crystallization events is obtained from 200 crystallization experiments with multiple sample preparations). Also, we confirmed the liquid structure at various supersaturation by multiple synchrotron experiments over a wide time span.
Data collection	Y.C.C. and L.W. conducted the statistical measurements of crystallization event. We continuously captured the sample images during experiments, then the supersaturation is derived from droplet volume calculated from images. Crystallization also can be distinguished from sample image due to shape deformation or transparency change. Accordingly, after repeating crystallization experiments (200 times for each sample), the probability distribution of the crystallization events for KDP and ADP is obtained. Y.C.C., S. Lee, L.W., Y.H.L., S.K., H.H.L., J.L. and G.W.L. performed synchrotron x-ray scattering experiments. The ESL is installed at Pohang Light Source II (PLS 5A and 1C beamlines) for the experiments. During experiments, monochromatized 18-KeV X-rays from a cryogenically cooled silicon (111) double crystal monochromator was delivered to the sample through a delivery pipe filed with helium gas. The x-ray detector (Pilatus-300 KW, 1475 x 195 pixels with a pixel size of 172 um x 172 um) was just placed behind the ESL chamber to collect the scattered signals from the solution droplet.

Timing and spatial scale	We started collecting droplet images after levitating the solution sample with initial supersaturation. Through these images, we can know the real-time supersaturation change with evaporation. Thus the supersaturation calculated from the sample image before crystallization is defined as the supersaturation for crystallization. To obtain the liquid structure at a certain supersaturation, we evaporated the sample droplets to the target supersaturation, then shoot the sample X-ray and record the signal with Pilatus-300 KW detector.
Data exclusions	No data were excluded from the analyses
Reproducibility	Statistical measurements are employed, no matter for crystallization events or synchrotron X-ray scattering experiments. These experiments are conducted with multiple experimental sample preparations over a wide time span. They give consistent experimental results.
Randomization	To eliminate the artificial effect, we test experimental samples prepared by different people, also the experiments are conducted by different people for cross-checking. In addition, experiments are performed under different periods to confirm the results.
Blinding	Though the blinding may be important to psychology research, it has no influence on the core objectives and outcomes in our liquid structural evolution and phase selection study.

Did the study involve field work? ☐ Yes ☒ No

Reporting for specific materials, systems and methods

We require information from authors about some types of materials, experimental systems and methods used in many studies. Here, indicate whether each material, system or method listed is relevant to your study. If you are not sure if a list item applies to your research, read the appropriate section before selecting a response.

Materials & experimental systems

n/a	Involved in the study
☒	☐ Antibodies
☒	☐ Eukaryotic cell lines
☒	☐ Palaeontology and archaeology
☒	☐ Animals and other organisms
☒	☐ Clinical data
☒	☐ Dual use research of concern
☒	☐ Plants

Methods

n/a	Involved in the study
☒	☐ ChIP-seq
☒	☐ Flow cytometry
☒	☐ MRI-based neuroimaging

Plants

Seed stocks	*Report on the source of all seed stocks or other plant material used. If applicable, state the seed stock centre and catalogue number. If plant specimens were collected from the field, describe the collection location, date and sampling procedures.*
Novel plant genotypes	*Describe the methods by which all novel plant genotypes were produced. This includes those generated by transgenic approaches, gene editing, chemical/radiation-based mutagenesis and hybridization. For transgenic lines, describe the transformation method, the number of independent lines analyzed and the generation upon which experiments were performed. For gene-edited lines, describe the editor used, the endogenous sequence targeted for editing, the targeting guide RNA sequence (if applicable) and how the editor was applied.*
Authentication	*Describe any authentication procedures for each seed stock used or novel genotype generated. Describe any experiments used to assess the effect of a mutation and, where applicable, how potential secondary effects (e.g. second site T-DNA insertions, mosiacism, off-target gene editing) were examined.*
